# Prenatal Exposure to Traffic-Related Air Pollution and Ultrasound Measures of Fetal Growth in the INMA Sabadell Cohort

**DOI:** 10.1289/ehp.0901228

**Published:** 2010-01-26

**Authors:** Inmaculada Aguilera, Raquel Garcia-Esteban, Carmen Iñiguez, Mark J. Nieuwenhuijsen, Àgueda Rodríguez, Montserrat Paez, Ferran Ballester, Jordi Sunyer

**Affiliations:** 1 Centre for Research in Environmental Epidemiology, Barcelona, Spain; 2 Municipal Institute of Medical Research (IMIM-Hospital del Mar), Barcelona, Spain; 3 Centro de Investigación Biomédica en Red de Epidemiología y Salud Pública, Barcelona, Spain; 4 Pompeu Fabra University, Barcelona, Spain; 5 Centre for Public Health Research, Conselleria de Sanitat, Generalitat Valenciana, Valencia, Spain; 6 Servei de Ginecologia i Obstetrícia, Hospital Parc Taulí, Sabadell, Spain; 7 Centre d’Atenció Primària Sant Fèlix, Sabadell, Spain

**Keywords:** air pollution, aromatic hydrocarbons, cohort study, exposure assessment, fetal growth, INMA study, land use regression, nitrogen dioxide, ultrasonography

## Abstract

**Background:**

Few studies have used longitudinal ultrasound measurements to assess the effect of traffic-related air pollution on fetal growth.

**Objective:**

We examined the relationship between exposure to nitrogen dioxide (NO_2_) and aromatic hydrocarbons [benzene, toluene, ethylbenzene, *m*/*p*-xylene, and *o*-xylene (BTEX)] on fetal growth assessed by 1,692 ultrasound measurements among 562 pregnant women from the Sabadell cohort of the Spanish INMA (Environment and Childhood) study.

**Methods:**

We used temporally adjusted land-use regression models to estimate exposures to NO_2_ and BTEX. We fitted mixed-effects models to estimate longitudinal growth curves for femur length (FL), head circumference (HC), abdominal circumference (AC), biparietal diameter (BPD), and estimated fetal weight (EFW). Unconditional and conditional SD scores were calculated at 12, 20, and 32 weeks of gestation. Sensitivity analyses were performed considering time–activity patterns during pregnancy.

**Results:**

Exposure to BTEX from early pregnancy was negatively associated with growth in BPD during weeks 20–32. None of the other fetal growth parameters were associated with exposure to air pollution during pregnancy. When considering only women who spent < 2 hr/day in nonresidential outdoor locations, effect estimates were stronger and statistically significant for the association between NO_2_ and growth in HC during weeks 12–20 and growth in AC, BPD, and EFW during weeks 20–32.

**Conclusions:**

Our results lend some support to an effect of exposure to traffic-related air pollutants from early pregnancy on fetal growth during mid-pregnancy.

Children are likely to be one of the most vulnerable populations to air pollution, particularly traffic-related pollutants (Schwartz 2004). Perhaps less expectedly, numerous studies in the last decade have reported that adverse effects of traffic-related air pollution manifest during the prenatal period by increasing the risk of intrauterine growth restriction (IUGR), low birth weight (LBW), and preterm birth, even at low air pollution levels (reviewed by Glinianaia et al. 2004; Lacasaña et al. 2005; Maisonet et al. 2004; Šrám et al. 2005). Results from these studies show considerable evidence for some birth outcomes (particularly IUGR and LBW) but are inconclusive in identifying the most harmful pollutants and the most susceptible periods during gestation. Heterogeneity in these findings may be explained in part by differences in study design, air exposure assessment, adjustment for confounding, and definition of birth outcomes (Woodruff et al. 2009).

Given the ubiquity of air pollution exposure and the importance of fetal growth as an indicator of children’s health, which may be associated with the development of chronic diseases in adulthood (Sinclair et al. 2007), more research is needed to disentangle the effects of individual pollutants, to understand the underlying biological mechanisms, and to identify specific periods of pregnancy when fetal growth may be more susceptible to air pollution effects (Slama et al. 2008a). Regarding exposure assessment, the use of spatiotemporal modeling approaches based on geographic information systems (GIS) and supported by subject-derived questionnaire data are encouraged (Gilliland et al. 2005).

To date, most of the studies investigating the relationship between prenatal exposure to air pollution and fetal growth have relied on neonatal anthropometric measurements as proxies of fetal growth, particularly birth weight (Brauer et al. 2008; Choi et al. 2008; Gouveia et al. 2004; Ha et al. 2001; Liu et al. 2007; Mannes et al. 2005; Parker et al. 2005; Rich et al. 2009; Slama et al. 2007; Wilhelm and Ritz 2005) and, to a lesser extent, birth height and head circumference (HC) at birth in addition to birth weight (Choi et al. 2006; Hansen et al. 2007; Jedrychowski et al. 2004). One limitation of these studies is that they are unable to assess fetal growth patterns. In addition, it has been suggested that birth weight poorly reflects IUGR during the first two trimesters of pregnancy (Hemachandra and Klebanoff 2006). Because different patterns of fetal growth and exposures may result in the same neonatal anthropometric measurement, studies using longitudinal ultrasound measurements may be helpful for identifying specific critical periods for the effect of air pollution on fetal growth (Hansen et al. 2008; Slama et al. 2009).

We previously reported an association between prenatal exposure to traffic-related air pollution (particularly during the second trimester of pregnancy) and birth weight in an urban cohort of pregnant women within the Spanish INMA (Environment and Childhood) study (Aguilera et al. 2009). Here we aim to investigate the relationship between prenatal exposure to air pollution and longitudinally measured fetal growth characteristics in the same cohort.

## Methods

### Study population

The INMA study is a multicenter population-based mother-and-child cohort study established in seven areas in Spain. The Sabadell cohort (Catalonia, Spain) comprises 657 pregnant women who were recruited at their first routine prenatal care visit in the primary care center between June 2004 and July 2006. Of the total sample of pregnant women, 93% (*n* = 611) lived in Sabadell at the beginning of the study, and 7% (*n* = 46) lived in two adjacent towns (Sant Quirze del Vallès and Barberà del Vallès) covered by the health service of the hospital of Sabadell. Main exclusion criteria were being < 16 years of age, having a nonsingleton pregnancy, not planning to deliver at the Sabadell hospital, and participating in an assisted reproduction program (Ribas-Fitó et al. 2006). Information on education, socioeconomic background, maternal health and obstetric history, parity, medication use, dietary intake, active and passive smoking, and time–activity patterns during pregnancy were obtained through questionnaires administered at weeks 12 and 32 of pregnancy. All participating women signed written informed consent forms, and the study was approved by the ethical committees of the Municipal Institute of Medical Research and the Hospital of Sabadell.

For this study, we restricted our analysis to women who were followed until the child’s birth, had at least two ultrasound measurements during pregnancy, and lived in Sabadell during the entire pregnancy (*n* = 562).

### Ultrasound measurements and fetal growth models

Routine fetal ultrasound examinations were conducted in early, middle, and late pregnancy both in the primary care center (first- and third-trimester ultrasounds) and in Sabadell hospital (second-trimester ultrasound). Fetal parameters recorded (in millimeters) were femur length (FL), HC, abdominal circumference (AC), and biparietal diameter (BPD). Estimated fetal weight (EFW) was calculated using the Hadlock algorithm (Hadlock et al. 1985). Because FL, HC, and AC are not routinely measured in the first trimester of pregnancy, ecographists from both centers were trained to follow the same protocol before the study start.

Gestational age was estimated using the date of the last menstrual period (LMP) reported at recruitment and confirmed by the first ultrasound examination. For women with ≥ 7 days’ difference between reported LMP and ultrasound-based LMP (*n* = 89; 16%), crown–rump length was used for establishing gestational age (Westerway et al. 2000).

We fitted extended mixed-effects models (Pinheiro and Bates 2000) to estimate longitudinal growth curves for the five fetal parameters using R software (version 2.6.0; R Project for Statistical Computing, Vienna, Austria). To obtain normally distributed outcomes at each gestational age with homoskedastic residual error variance, we first estimated the power transformation of each of the fetal outcomes by modeling their transformed mean as a cubic polynomial in gestational age (in days), using the “boxcox” function from the main package of Venables and Ripley’s MASS library (Royston 1995).

We used generalized least squares (gls function from the nlme library) to fit growth models with heteroskedastic (i.e., with unequal variances) and/or correlated within-subject errors but with no random effects. Variable selection using Akaike information criterion was carried out through a forward-selection procedure on covariates considered to be determinants either of size or growth: child’s sex, maternal age, parity, ethnicity, height and prepregnancy weight, and paternal height and weight, and their interactions with gestational age. Covariates were retained in the model if the likelihood ratio test *p*-value was < 0.10. Linear terms, quadratic terms, and tertiles of each continuous covariate were considered. Then we analyzed correlation structures to model the dependence among the within-subject errors. Next, we assessed heteroskedasticity by estimating the variance of the errors as a function of child’s sex, gestational age, and indicator variables tagging pregnancies with at least two consecutive ultrasounds performed within short time intervals (18, 21, and 30 days). Finally, random effects at intercept or/and slope (lme function) were tested using likelihood ratio tests and residual diagnosis (autocorrelation function). Growth models for the five fetal parameters are shown in the Supplemental Material (doi:10.1289/ehp.0901228).

Growth models were applied to calculate unconditional SD scores at 12, 20, and 32 weeks of gestation, and conditional SD scores over the week intervals 12–20 and 20–32. These three cutoff weeks represent the most common schedule for the three routine ultrasounds in the Spanish prenatal care system. Unconditional SD scores represent cross-sectional estimates of fetal size, whereas conditional SD scores represent estimates of fetal growth because they take into account the fetal size earlier in gestation, which is a determinant of subsequent fetal growth (Owen et al. 2000; Royston 1995).

For the two fetal parameters that were directly measured both by ultrasound examination and at birth (HC and AC), we calculated the intraclass correlation coefficient (ICC) to assess agreement between the predictions and the measures at birth. The ICCs for HC and AC were 0.57 and 0.63, respectively (*p* < 0.01).

### Air pollution exposure assessment

Land-use regression (LUR) modeling was used to estimate individual exposure to traffic-related air pollution in the cohort (Aguilera et al. 2008). We chose this GIS-based technique because of its ability to capture small-scale variations in air pollution levels within urban areas (Briggs et al. 2000). Briefly, passive samplers were used to measure nitrogen dioxide (NO_2_) and BTEX (benzene, toluene, ethylbenzene, *m*/*p*-xylene, and *o*-xylene) as markers of motor vehicle exhaust. One-week measurements were carried out at 57 sampling sites in four and three sampling campaigns for NO_2_ and BTEX, respectively. Concentrations of all the sampling campaigns were averaged to represent annual mean levels of each pollutant (Lebret et al. 2000), and linear regression models were fitted for NO_2_ (*R*^2^ = 0.75) and BTEX (*R*^2^ = 0.74) using five groups of geographic characteristics (land coverage, topography, population density, roads, and distance to local sources of pollution) as predictor variables. Models were then applied to predict outdoor air pollution levels at the cohort addresses, accounting for different home addresses for those women who moved within Sabadell during pregnancy (*n* = 25). For budgetary reasons, we were not able to perform air pollution measurements in the two adjacent towns of Sant Quirze del Vallès and Barberà del Vallès, so LUR estimates were not available for the 46 women who lived in these two cities.

To calculate individual exposures to air pollutants during specific periods of pregnancy, both LUR models were adjusted for temporal variations of daily NO_2_ levels measured in the fixed monitoring station of Sabadell, assuming similar temporal variations in NO_2_ and BTEX levels (Aguilera et al. 2009). Using this procedure, for each woman we calculated average cumulative exposures to NO_2_ and BTEX from the LMP up to 12, 20, and 32 weeks of pregnancy, as well as average exposures during weeks 12–20 and weeks 20–32. These five windows of exposure were chosen to be comparable to the periods when fetal size and fetal growth were estimated using mixed-effects models.

### Statistical analysis

Statistical analyses were conducted using STATA (version 10.1; StataCorp LP, College Station, TX, USA). Associations between unconditional and conditional SD scores and levels of exposure to NO_2_ and BTEX were examined by simple and multiple linear regression models. To be able to compare these results with our previous study on air pollution and birth weight within the same cohort (Aguilera et al. 2009), we used the LUR estimate of the sum of the five BTEX compounds in the statistical analysis. For the same reason, we adjusted the associations for the same covariates as those included in our previous study, after examining for potential collinearity: season of conception, child’s sex, maternal age, maternal education, maternal ethnicity, parity, smoking during pregnancy, maternal height and prepregnancy weight, and paternal height and weight. However, covariates already included in each mixed-effects model were not considered again for adjustment in the multivariate analysis.

Because in our previous study we found more pronounced associations between prenatal exposure to air pollution and birth weight among two subsets of women potentially less prone to exposure misclassification due to some specific time–activity patterns during pregnancy (Aguilera et al. 2009), as a sensitivity analysis here we also examined the associations for the same two subsets: women who spent ≥ 15 hr/day at home (*n* = 274) and women who spent < 2 hr/day in nonresidential outdoor environments (*n* = 255), the two cutoffs being the median of the distribution of each variable. Both time–activity variables were reported at the third trimester of pregnancy and were meant to represent a typical week during pregnancy. Because exposure estimates were residence based, we assumed that these two subsets suffered less from exposure misclassification.

Finally, we compared the results obtained using SD scores from growth models with those from linear regression models developed for each trimester of pregnancy. In this cross-sectional analysis, we used the exposure windows during weeks 1–12, 12–20, and 20–32 to assess their relationship with the fetal parameters recorded in the first, second, and third trimester, respectively, and we adjusted the associations for the same covariates as in the main analysis.

## Results

Table 1 shows the characteristics of the study population. Most women were nulliparous (56.6%), nonsmokers during the entire pregnancy (68.2%), and exposed to passive smoking either at home or at work (53.7%) and had at least secondary education (70.7%).

A total of 1,692 ultrasound examinations were performed for the 562 pregnancies (Table 2). Most women had one routine ultrasound examination during each trimester of pregnancy (*n* = 556); however, 17 women (3%) had four to six examinations.

Table 3 provides the distribution of exposures to NO_2_ and BTEX during specific periods of pregnancy and the Spearman correlation coefficients among them. We found only slight differences among mean levels of both pollutants by exposure interval. Between-period correlation coefficients were higher among BTEX exposures (*r* = 0.71–0.73) than among NO_2_ exposures (*r* = 0.46–0.52). Mean cumulative exposures during weeks 1–20 (NO_2_, 32.1 μg/m^3^; BTEX, 14.7 μg/m^3^) and during weeks 1–32 (NO_2_, 32.0 μg/m^3^; BTEX, 14.7 μg/m^3^) were very similar to mean exposures during weeks 1–12 and highly correlated for both NO_2_ (*r* = 0.81–0.89) and BTEX (*r* = 0.91–0.96).

The unadjusted mean change in SD scores of fetal size (at weeks 12, 20, and 32) and fetal growth (during weeks 12–20 and weeks 20–32) for an interquartile range (IQR) increase in exposure to NO_2_ and BTEX during weeks 1–12, 12–20, and 20–32 showed that exposure to both NO_2_ and BTEX during weeks 1–12 was negatively associated with growth in BPD between weeks 20 and 32 of pregnancy (NO_2_, β = −0.075, *p* = 0.03; BTEX, β = −0.124, *p* = 0.01). Moreover, cumulative exposure during weeks 1–20 was associated with the same outcome (data not shown), but the exposure during weeks 12–20 was not. We found an association between exposure to BTEX during weeks 1–12 and size in BPD at week 32 of pregnancy (β = −0.095, *p* = 0.05). None of the other fetal parameters was significantly associated with any of the exposure periods to air pollution. After adjustment for potential confounders, associations between NO_2_ and BTEX exposure during weeks 1–12 and growth in BPD during weeks 20–32 were very similar to the unadjusted associations but only statistically significant at the *p* < 0.05 level for BTEX (Table 4). We repeated the analysis removing the extra scans of the 17 women who had more than the three routine ultrasound measurements, but effect estimates did not differ substantially from those reported in Table 4.

When we restricted the analysis to women who spent ≥ 15 hr/day at home during pregnancy, we found stronger, not statistically significant, associations between BTEX exposure during weeks 1–12 and SD scores for most of the fetal parameters (Table 5). When considering only women who spent < 2 hr/day in nonresidential outdoor environments, we found consistently higher associations between NO_2_ exposure during weeks 1–12 and all the SD scores, which reached statistical significance (*p* < 0.05) for growth in HC between weeks 12 and 20, growth in AC, BPD, and EFW between weeks 20 and 32, size in HC, AC, and EFW at week 32, and size in HC at week 20. Associations for BTEX exposure during the same period were also stronger but statistically significant only for the same outcomes as in the whole cohort. Given their high correlation with exposure during weeks 1–12, cumulative exposures during weeks 1–20 and 1–32 showed similar associations in these two subsets (data not shown), but the exposure during weeks 12–20 and weeks 20–32 was not associated with any SD score.

Finally, results from the cross-sectional analysis showed no significant association between exposure to air pollution during the three exposure windows (weeks 1–12, 12–20, and 20–32) and the corresponding trimester-specific fetal parameters [see Supplemental Material, Table 1 (doi:10.1289/ehp.0901228)].

## Discussion

In this cohort of pregnant women from Sabadell, Spain, we found an association between exposure to traffic-related air pollution from the beginning of the pregnancy and impaired growth in BPD during mid- to late pregnancy. None of the other fetal growth characteristics was associated with exposure to air pollution in any of the periods studied for the whole cohort. The magnitude of most of the associations for BTEX exposure was more pronounced, although not statistically significant, among women who spent ≥ 15 hr/day at home, compared with the whole cohort. Only among women who spent < 2 hr/day in nonresidential outdoor environments were associations statistically significant. We found adverse effects of exposure to NO_2_ from the beginning of pregnancy on growth in HC during weeks 12–20 and growth in AC and EFW (in addition to BPD) during weeks 20–32. HC at 20 and 32 weeks and AC and EFW at week 32 were also associated with NO_2_ exposure in this subset.

This is the first study to use exposure assessment based on LUR models to investigate the effect of prenatal exposure to traffic-related air pollution on ultrasound-based fetal growth. So far, only two studies have assessed fetal growth by ultrasound measurements, using different exposure assessment approaches. Hansen et al. (2008) assigned air pollution data from the closest monitoring station to each woman’s residential postal code. They found an association between exposure to low levels of air pollution during early pregnancy and decreased fetal growth characteristics in mid-pregnancy, although they included only scans between weeks 13 and 26 of pregnancy and therefore were not able to study the effect of air pollution on fetal growth either in the first or the third trimester of pregnancy. Slama et al. (2009) assessed benzene exposure by using personal monitoring during 1 week and found an association with BPD at each of the trimester ultrasound examinations and with HC at the second- and third-trimester ultrasound examinations. Although we used a different approach than these two studies in terms of exposure assessment and statistical analysis, our results lend some support to an effect of air pollution exposure on fetal growth starting at mid-pregnancy. However, results were irregular and did not show a clear pattern in relation to the different fetal characteristics. In addition, the high correlations among the measured fetal parameters (particularly during the first and second trimester of pregnancy) make the interpretation of the results difficult in terms of which one is most affected by air pollution exposure.

Exposure to a specific environmental factor in early, middle, and late pregnancy is likely to affect the fetus differently. In addition, according to the fetal programming hypothesis, the timing when an adverse effect occurs as a result of the exposure is crucial in determining the risk for diseases during adulthood (Nathanielsz 2000). The second trimester is the period of maximal growth velocity of the placenta, so an abnormal pattern of placental growth earlier in gestation may result in abnormal fetal growth during middle or late pregnancy and lead to an IUGR newborn (Lestou and Kalousek 1998). One of the proposed biological mechanisms by which air pollution may affect fetal growth is by binding receptors for placental growth factors and consequently decreasing placental–fetal exchange of oxygen and nutrients (Kannan et al. 2006). If so, this could explain the influence of exposure from early pregnancy on fetal growth during mid-pregnancy.

An accurate assessment of air pollution exposure is particularly important in studies on reproductive outcomes, where the exposure period is clearly defined and there is concern about the existence of potential windows of susceptibility. If exposure assessment is based on residential location during pregnancy, the extent to which air pollution levels around the residence represent personal exposure will depend on several factors, including activity and mobility patterns during the exposure period (Nethery et al. 2009). Therefore, sensitivity analyses taking into account differences in time–activity patterns (e.g., residential and occupational mobility, work status, time spent at or near home) are needed to verify the impact of exposure misclassification on effect estimates (Ritz and Wilhelm 2008). In a previous study (Aguilera et al. 2009) we found an association between exposure to NO_2_ and BTEX during the second trimester of pregnancy on birth weight, but it was statistically significant only among women who spent < 2 hr/day in nonresidential outdoor environments. Given that we had based LUR estimates in women’s residential addresses, we argued that women spending ≥ 2 hr/day in nonresidential outdoor were potentially more exposed to different levels of traffic-related air pollutants not reflected by the residence-based LUR estimates and therefore were more prone to exposure misclassification. The stronger effects found among the same subset of women for most of the fetal parameters in the present study are also in accordance with this hypothesis.

Because we estimated levels of NO_2_ and BTEX with LUR models (which account for small-scale variability in concentrations of traffic-related pollutants), we considered these pollutants as markers of vehicle exhaust toxins rather than potential causative agents by themselves. In our previous study on air pollution and birth weight we found consistently stronger associations for BTEX than for NO_2_, but in the present study none clearly emerged as a potentially better marker of altered fetal growth due to exposure to traffic-related air pollution (although results from the previous and the present study cannot be interpreted as fully independent). Overall, the high correlation in space and time between pollutants sharing similar sources, together with the lack of enough knowledge on underlying casual pathways, makes it difficult to separate the etiologic agents and to disentangle the role of independent pollutants in causing adverse health effects (Kim et al. 2007).

It is important to distinguish between size and growth when attempting to identify IUGR fetuses. Conditional SD scores, which take into account earlier measures of fetal size, are more appropriate for assessing fetal growth and identifying IUGR than using cross-sectional estimates of fetal size (Owen et al. 2000; Royston 1995). This difference between conditional and unconditional SD scores may explain why an association between air pollution exposure and SD scores of fetal size at two different weeks do not imply an association for SD scores of fetal growth between these two weeks (as shown in Table 5 for HC).

One of the strengths of our study is that it is a population-based cohort followed from early pregnancy onward, with information on many potential confounders at individual levels and well-controlled data quality. In addition, we estimated prenatal exposure to air pollution using temporally adjusted LUR models applied to geocoded residential addresses and accounting for residential mobility during pregnancy.

We established gestational age from the date of the LMP and corrected those cases that differed by ≥ 7 days with the estimate of crown–rump length obtained in the first trimester ultrasound. This may underestimate potential effects of air pollution if exposure shows an early effect on fetal growth (Slama et al. 2008b). However, we expect this effect would have been small in our results for two reasons: *a*) crown–rump length was not associated with early exposure to air pollution, and *b*) conditional SD scores reflect change in size and therefore are unlikely to have been affected by gestational age error (Pedersen et al. 2008).

One concern about using ultrasound measurements to assess the effects of any exposure of interest on fetal growth is measurement error. Potential measurement errors in clinical practice include the use of different ultrasound units and interobserver variability (Perni et al. 2004). In our cohort, the ultrasound examinations were carried out in two centers for all the women, which limited the number of ultrasound units and ecographists performing the measurements. In addition, we used multiple observations per fetus and modeled each fetus against the average curve, which should have reduced the measurement error.

One limitation of our study is that we did not account for indoor exposures to air pollution or for factors affecting the influence of outdoor pollution on indoor environments (e.g., air conditioning). Some traffic-related air pollutants (e.g., NO_2_, particulate matter, polycyclic aromatic hydrocarbons, or volatile organic compounds) have also relevant indoor sources that could contribute to important interindividual variations in exposure, although the limited data about the identification of the most harmful pollutants and their biological pathways do not allow us to evaluate the real importance of indoor exposure on the relationship between air pollution and fetal growth.

A second limitation is that we performed 170 comparisons between exposures and outcomes, which may have led to spurious findings. Finally, because of the small number of ultrasound measurements performed from week 35 onward, we could not assess the influence of air pollution exposure on fetal growth during late pregnancy (i.e., weeks 32–38), when most of the constitutional variation in fetal parameters occurs (Hindmarsh et al. 2002).

## Conclusions

We found an effect of prenatal exposure to urban air pollution on growth in BPD between weeks 20 and 32 of gestation. Among women who spent < 2 hr/day in nonresidential outdoor locations, associations were stronger and statistically significant for growth in HC during weeks 12–20 and growth in AC, BPD, and EFW during weeks 20–32. Overall, air pollution exposure from early pregnancy seems to affect fetal growth during mid-pregnancy. Sensitivity analysis using time–activity patterns during pregnancy should be performed to examine potential variations in effect estimates. We found no consistently higher associations with impaired fetal growth for either NO_2_ or BTEX, taken as markers of a complex mixture of vehicle exhaust toxins.

## Figures and Tables

**Table 1:** Characteristics of the study population (*n* = 562).

Characteristic	*n*	Percent
Child’s sex		
Male	286	50.9
Female	276	49.1
Season of conception		
Spring	156	27.8
Summer	165	29.4
Fall	129	23.0
Winter	112	19.9
Smoking during pregnancy		
Never	231	42.0
Former, quit before pregnancy	144	26.2
Former, quit during pregnancy	84	15.3
Current	91	16.6
Passive smoking (reference: no)		
At home	197	35.8
At work	149	27.0
Either at home or at work	296	53.7
Maternal education		
Primary education	164	29.3
Secondary education	233	41.7
University degree	162	29.0
Maternal race/ethnicity		
White/Caucasian	543	96.8
Latin American	14	2.5
Black	4	0.7
Child birth order		
First	317	56.6
Second	206	36.8
Third or more	37	6.6
Continuous variable	Mean ± SD	Range

Maternal age (years)	31.0 ± 4.3	17–43
Gestational age (weeks)	39.7 ± 1.4	34–42
Maternal height (cm)	162.4 ± 6.1	146–180
Maternal prepregnancy weight (kg)	62.6 ± 12.8	39–143
Paternal height (cm)	175.9 ± 7.1	150–197
Paternal weight (kg)	80.2 ± 12.5	50–130
Birth weight (g)	3247.4 ± 423.4	1,750–4,480
Birth height (cm)	49.4 ± 1.9	44–55
Birth HC (cm)	34.2 ± 1.2	30–38
Birth AC (cm)	31.3 ± 2.0	25–37

**Table 2:** Descriptive statistics of ultrasound measurements (*n* = 1,692).

Ultrasound	Gestational age (weeks)	Fetal characteristics (mm)			
		FL	HC	AC	BPD
First					
No. of scans	—	512	512	498	553
Mean ± SD	12.2 ± 1.0	7.8 ± 2.5	75.3 ± 12.8	62.1 ± 11.5	20.7 ± 3.7
Second					
No. of scans	—	560	556	560	561
Mean ± SD	21.1 ± 1.0	35.0 ± 3.3	189.1 ± 13.9	164.0 ± 14.2	49.5 ± 3.8
Third					
No. of scans	—	555	554	554	553
Mean ± SD	34.0 ± 1.3	66.1 ± 3.2	309.2 ± 13.3	300.0 ± 16.2	86.1 ± 3.8
Fourth					
No. of scans	—	17	17	17	17
Mean ± SD	35.1 ± 3.0	66.9 ± 6.5	318.3 ± 19.5	306.9 ± 28.8	86.7 ± 7.1
Fifth					
No. of scans	—	2	2	2	2
Mean ± SD	33.7 ± 3.2	67.5 ± 10.6	331.5 ± 17.7	305.5 ± 20.5	85.5 ± 7.8
Sixth					
No. of scans	—	1	1	0	1
Mean ± SD	34.4 ± 0	64.6 ± 0	342 ± 0	—	85.8 ± 0

**Table 3:** Descriptive statistics of NO_2_ and BTEX exposure periods (μg/m^3^) and Spearman correlation coefficients between different exposure periods

				Spearman correlation coefficients*		
Pollutant/exposure period	Mean ± SD	Range	IQR	Weeks 1–12	Weeks 12–20	Weeks 20–32
NO_2_						
Weeks 1–12	32.45 ± 10.51	8.91–76.16	12.19	1		
Weeks 12–20	31.68 ± 10.81	6.65–75.74	11.47	0.52	1	
Weeks 20–32	32.13 ± 10.84	9.83–73.01	13.23	0.46	0.48	1

BTEX						
Weeks 1–12	14.89 ± 6.24	2.27–30.31	9.66	1		
Weeks 12–20	14.47 ± 6.15	1.99–31.83	9.99	0.73	1	
Weeks 20–32	14.72 ± 6.30	2.79–28.90	10.19	0.72	0.71	1

* *p* < 0.01 for all correlation coefficients.

**Table 4:** Adjusted^a^ mean percent change (95% confidence interval) in SD scores of fetal size and growth for an IQR increase in exposure during different periods of pregnancy: full cohort.

	Period of exposure during pregnancy					
	Weeks 1–12		Weeks 12–20		Weeks 20–32	
Characteristic/week	NO_2_	BTEX	NO_2_	BTEX	NO_2_	BTEX
FL						
Week 12	−1.32 (−4.54 to 1.91)	−0.76 (−5.08 to 3.57)	NA	NA	NA	NA
Week 20	−0.70 (−4.3 to 2.91)	−2.16 (−6.97 to 2.68)	−1.03 (−4.3 to 2.25)	−3.14 (−8.06 to 1.82)	NA	NA
Week 32	−0.28 (−3.2 to 2.64)	0.18 (−3.74 to 4.1)	1.54 (−1.11 to 4.18)	1.97 (−2.06 to 5.97)	0.23 (−2.86 to 3.32)	0.21 (−3.88 to 4.29)
Weeks 12–20	−0.23 (−3.79 to 3.34)	−2.02 (−6.78 to 2.76)	−1.27 (−4.5 to 1.97)	−3.48 (−8.34 to 1.43)	NA	NA
Weeks 20–32	−0.12 (−3.04 to 2.79)	0.68 (−3.23 to 4.59)	1.81 (−0.84 to 4.44)	2.73 (−1.29 to 6.71)	0.84 (−2.25 to 3.91)	1.25 (−2.83 to 5.32)

HC						
Week 12	0.16 (−3.51 to 3.83)	−0.08 (−4.99 to 4.83)	NA	NA	NA	NA
Week 20	−1.44 (−5.1 to 2.24)	−0.36 (−5.27 to 4.56)	1.04 (−2.25 to 4.31)	1.4 (−3.6 to 6.37)	NA	NA
Week 32	−0.92 (−3.96 to 2.13)	0.40 (−3.66 to 4.46)	0.76 (−1.98 to 3.49)	1.91 (−2.23 to 6.04)	0.16 (−3.04 to 3.36)	1.64 (−2.66 to 5.91)
Weeks 12–20	−1.68 (−5.26 to 1.92)	−0.36 (−5.19 to 4.48)	1.12 (−2.17 to 4.39)	1.91 (−3.01 to 6.81)	NA	NA
Weeks 20–32	−0.52 (−3.56 to 2.53)	0.52 (−3.62 to 4.65)	0.48 (−2.33 to 3.29)	1.56 (−2.66 to 5.76)	−0.04 (−3.32 to 3.24)	1.36 (−2.94 to 5.64)

AC						
Week 12	−1.83 (−5.34 to 1.68)	−1.91 (−6.58 to 2.77)	NA	NA	NA	NA
Week 20	−0.48 (−4.22 to 3.27)	−1.16 (−6.14 to 3.84)	−0.08 (−3.44 to 3.28)	−1.6 (−6.72 to 3.56)	NA	NA
Week 32	−1.32 (−4.44 to 1.81)	−1.04 (−5.24 to 3.18)	−0.08 (−2.89 to 2.73)	−0.16 (−4.45 to 4.13)	0.52 (−2.76 to 3.80)	0.56 (−3.81 to 4.92)
Weeks 12–20	0.44 (−3.31 to 4.18)	−0.32 (−5.31 to 4.67)	0.84 (−2.52 to 4.19)	−0.16 (−5.3 to 4.99)	NA	NA
Weeks 20–32	−1.24 (−4.36 to 1.89)	−0.72 (−4.85 to 3.42)	−0.04 (−2.85 to 2.77)	0.32 (−3.97 to 4.61)	0.84 (−2.44 to 4.11)	1.24 (−3.14 to 5.6)

BPD						
Week 12	2.51 (−1.32 to 6.32)	2.19 (−2.96 to 7.31)	NA	NA	NA	NA
Week 20	2.95 (−0.57 to 6.44)	2.39 (−2.30 to 7.05)	2.99 (−0.21 to 6.17)	1.83 (−3.01 to 6.65)	NA	NA
Week 32	−1.64 (−4.83 to 1.57)	−3.74 (−8.07 to 0.63)	0.00 (−2.97 to 2.97)	−1.83 (−6.27 to 2.62)	1.04 (−2.40 to 4.47)	−0.80 (−5.32 to 3.73)
Weeks 12–20	1.99 (−1.6 to 5.57)	1.52 (−3.33 to 6.34)	2.79 (−0.49 to 6.05)	1.91 (−3.09 to 6.88)	NA	NA
Weeks 20–32	−2.75 (−6.01 to 0.53)	−4.82 (−9.12 to −0.45)*	−1.04 (−4 to 1.93)	−2.59 (−7.01 to 1.86)	0.60 (−2.84 to 4.03)	−1.04 (−5.55 to 3.49)

EFW						
Week 12	−1.44 (−4.79 to 1.93)	−1.12 (−5.63 to 3.41)	NA	NA	NA	NA
Week 20	0.20 (−4.02 to 4.41)	−0.88 (−6.48 to 4.74)	0.28 (−3.55 to 4.1)	−1.83 (−7.58 to 3.94)	NA	NA
Week 32	−1.36 (−4.4 to 1.69)	−1.4 (−5.45 to 2.67)	0.6 (−2.14 to 3.33)	0.40 (−3.82 to 4.61)	0.64 (−2.57 to 3.84)	0.44 (−3.86 to 4.73)
Weeks 12–20	1.16 (−2.83 to 5.13)	−0.36 (−5.73 to 5.02)	0.6 (−3.07 to 4.27)	−1.40 (−6.91 to 4.15)	NA	NA
Weeks 20–32	−1.60 (−4.63 to 1.45)	−1.12 (−5.17 to 2.95)	0.52 (−2.22 to 3.25)	1.20 (−3.02 to 5.40)	1.28 (−1.93 to 4.47)	1.71 (−2.58 to 5.99)

NA, not applicable.

a All adjusted for season of conception, parity, maternal educational level, and maternal smoking. Models for BPD and FL also included maternal prepregnancy weight. The model for FL also included child’s sex.

* *p* < 0.05.

**Table 5:** Adjusted^a^ mean percent change (95% confidence interval) in SD scores of fetal size and growth for an IQR increase in exposure between weeks 1 and 12 of pregnancy: two time–activity subsets of the cohort.

	Women who spent ≥ 15 hr/day at home (*n* = 274)		Women who spent < 2 hr/day in nonresidential outdoor environments (*n* = 255)	
Characteristic/week	NO_2_	BTEX	NO_2_	BTEX
FL				
Week 12	−1.40 (−5.91 to 3.14)	−0.76 (−6.75 to 5.25)	−2.31 (−7.81 to 3.23)	−1.99 (−8.96 to 5.03)
Week 20	−3.35 (−8.52 to 1.89)	−4.22 (−11.04 to 2.73)	−3.19 (−8.98 to 2.67)	−3.27 (−10.57 to 4.15)
Week 32	1.75 (−2.62 to 6.11)	1.20 (−4.58 to 6.95)	−1.87 (−6.61 to 2.89)	1.75 (−4.34 to 7.80)
Weeks 12–20	−3.07 (−8.17 to 2.09)	−4.22 (−10.89 to 2.57)	−2.55 (−8.2 to 3.15)	−2.75 (−9.92 to 4.51)
Weeks 20–32	2.55 (−1.75 to 6.82)	2.15 (−3.55 to 7.81)	−1.20 (−5.94 to 3.57)	2.51 (−3.58 to 8.55)

HC				
Week 12	0.56 (−4.59 to 5.70)	−1.91 (−8.65 to 4.88)	−3.23 (−9.32 to 2.94)	−3.19 (−10.87 to 4.62)
Week 20	−3.39 (−8.64 to 1.93)	−2.67 (−9.62 to 4.36)	−7.02 (−12.78 to −1.12)*	−3.35 (−10.88 to 4.30)
Week 32	−0.36 (−4.80 to 4.09)	−1.91 (−7.81 to 4.02)	−5.41 (−10.24 to −0.5)*	−1.87 (−8.15 to 4.45)
Weeks 12–20	−4.02 (−9.19 to 1.21)	−2.03 (−8.99 to 4.99)	−6.24 (−11.94 to −0.4)*	−2.19 (−9.61 to 5.30)
Weeks 20–32	0.68 (−3.85 to 5.20)	−1.20 (−7.18 to 4.81)	−3.51 (−8.60 to 1.65)	−0.92 (−7.44 to 5.63)

AC				
Week 12	−3.11 (−8.21 to 2.05)	−2.67 (−9.39 to 4.12)	−4.26 (−10.18 to 1.75)	−2.63 (−10.26 to 5.09)
Week 20	0.76 (−4.78 to 6.28)	−1.20 (−8.56 to 6.21)	−2.07 (−8.27 to 4.17)	−3.35 (−11.1 to 4.54)
Week 32	−0.24 (−4.84 to 4.37)	−0.32 (−6.47 to 5.84)	−5.25 (−10.16 to −0.26)*	−3.51 (−9.82 to 2.90)
Weeks 12–20	2.47 (−3.15 to 8.05)	0.04 (−7.42 to 7.5)	−0.16 (−6.08 to 5.76)	−2.39 (−9.88 to 5.18)
Weeks 20–32	−0.52 (−5.27 to 4.24)	0.04 (−6.27 to 6.35)	−4.86 (−9.62 to −0.02)*	−2.59 (−8.78 to 3.66)

BPD				
Week 12	2.59 (−2.80 to 7.94)	1.99 (−5.18 to 9.11)	1.91 (−4.41 to 8.19)	3.07 (−4.97 to 10.98)
Week 20	3.27 (−1.50 to 7.99)	3.03 (−3.38 to 9.36)	4.89 (−0.57 to 10.26)	4.7 (−2.25 to 11.50)
Week 32	−2.11 (−7.08 to 2.89)	−4.62 (−11.13 to 2.02)	−3.43 (−8.6 to 1.81)	−5.45 (−11.93 to 1.18)
Weeks 12–20	2.31 (−2.69 to 7.28)	2.39 (−4.17 to 8.89)	4.54 (−1.24 to 10.22)	3.70 (−3.71 to 11.00)
Weeks 20–32	−3.39 (−8.41 to 1.69)	−5.96 (−12.5 to 0.74)	−5.37 (−10.65 to −0.01)*	−7.38* (−13.93 to −0.62)

EFW				
Week 12	−2.67 (−7.40 to 2.10)	−2.11 (−8.31 to 4.13)	−2.99 (−8.78 to 2.87)	−1.83 (−9.18 to 5.57)
Week 20	−0.12 (−6.27 to 6.03)	−1.83 (−9.86 to 6.27)	−1.71 (−8.68 to 5.31)	−2.59 (−11.42 to 6.37)
Week 32	−0.28 (−4.88 to 4.33)	−1.20 (−7.33 to 4.97)	−5.05 (−9.81 to −0.22)*	−3.19 (−9.28 to 2.98)
Weeks 12–20	1.56 (−4.46 to 7.53)	−0.84 (−8.82 to 7.18)	−0.12 (−6.66 to 6.42)	−1.91 (−10.17 to 6.42)
Weeks 20–32	−0.24 (−5.00 to 4.52)	−0.52 (−6.82 to 5.79)	−4.78 (−9.47 to −0.02)*	−2.39 (−8.43 to 3.70)

a All adjusted for season of conception, parity, maternal educational level, and maternal smoking. Models for BPD and FL also included maternal prepregnancy weight. The model for FL also included child’s sex.

* *p* < 0.05.

## References

[b1-ehp-118-705] Aguilera I, Guxens M, Garcia-Esteban R, Corbella T, Nieuwenhuijsen M, Foradada CM (2009). Association between GIS-based exposure to urban air pollution during pregnancy and birth weight in the INMA Sabadell cohort. Environ Health Perspect.

[b2-ehp-118-705] Aguilera I, Sunyer J, Fernández-Patier R, Hoek G, Aguirre-Alfaro A, Meliefste K (2008). Estimation of outdoor NO_x_, NO_2_, and BTEX exposure in a cohort of pregnant women using land use regression modeling. Environ Sci Technol.

[b3-ehp-118-705] Brauer M, Lencar C, Tamburic L, Koehoorn M, Demers P, Karr C (2008). A cohort study of traffic-related air pollution impacts on birth outcomes. Environ Health Perspect.

[b4-ehp-118-705] Briggs DJ, de Hoogh C, Gulliver J, Wills J, Elliott P, Kingham S (2000). A regression-based method for mapping traffic-related air pollution: application and testing in four contrasting urban environments. Sci Total Environ.

[b5-ehp-118-705] Choi H, Jedrychowski W, Spengler J, Camann DE, Whyatt RM, Rauh V (2006). International studies of prenatal exposure to polycyclic aromatic hydrocarbons and fetal growth. Environ Health Perspect.

[b6-ehp-118-705] Choi H, Rauh V, Garfinkel R, Tu Y, Perera FP (2008). Prenatal exposure to airborne polycyclic aromatic hydrocarbons and risk of intrauterine growth restriction. Environ Health Perspect.

[b7-ehp-118-705] Gilliland F, Avol E, Kinney P, Jerrett M, Dvonch T, Lurmann F (2005). Air pollution exposure assessment for epidemiologic studies of pregnant women and children: lessons learned from the Centers for Children’s Environmental Health and Disease Prevention Research. Environ Health Perspect.

[b8-ehp-118-705] Glinianaia SV, Rankin J, Bell R, Pless-Mulloli T, Howel D (2004). Particulate air pollution and fetal health: a systematic review of the epidemiologic evidence. Epidemiology.

[b9-ehp-118-705] Gouveia N, Bremner SA, Novaes HM (2004). Association between ambient air pollution and birth weight in São Paulo, Brazil. J Epidemiol Community Health.

[b10-ehp-118-705] Ha EH, Hong YC, Lee BE, Woo BH, Schwartz J, Christiani DC (2001). Is air pollution a risk factor for low birth weight in Seoul?. Epidemiology.

[b11-ehp-118-705] Hadlock FP, Harrist RB, Sharman RS, Deter RL, Park SK (1985). Estimation of fetal weight with the use of head, body, and femur measurements—a prospective study. Am J Obstet Gynecol.

[b12-ehp-118-705] Hansen C, Neller A, Williams G, Simpson R (2007). Low levels of ambient air pollution during pregnancy and fetal growth among term neonates in Brisbane, Australia. Environ Res.

[b13-ehp-118-705] Hansen CA, Barnett AG, Pritchard G (2008). The effect of ambient air pollution during early pregnancy on fetal ultrasonic measurements during mid-pregnancy. Environ Health Perspect.

[b14-ehp-118-705] Hemachandra AH, Klebanoff MA (2006). Use of serial ultrasound to identify periods of fetal growth restriction in relation to neonatal anthropometry. Am J Hum Biol.

[b15-ehp-118-705] Hindmarsh PC, Geary MP, Rodeck CH, Kingdom JC, Cole TJ (2002). Intrauterine growth and its relationship to size and shape at birth. Pediatr Res.

[b16-ehp-118-705] Jedrychowski W, Bendkowska I, Flak E, Penar A, Jacek R, Kaim I (2004). Estimated risk for altered fetal growth resulting from exposure to fine particles during pregnancy: an epidemiologic prospective cohort study in Poland. Environ Health Perspect.

[b17-ehp-118-705] Kannan S, Misra DP, Dvonch JT, Krishnakumar A (2006). Exposures to airborne particulate matter and adverse perinatal outcomes: a biologically plausible mechanistic framework for exploring potential effect modification by nutrition. Environ Health Perspect.

[b18-ehp-118-705] Kim JY, Burnett RT, Neas L, Thurston GD, Schwartz J, Tolbert PE (2007). Panel discussion review: session two—interpretation of observed associations between multiple ambient air pollutants and health effects in epidemiologic analyses. J Expo Sci Environ Epidemiol.

[b19-ehp-118-705] Lacasaña M, Esplugues A, Ballester F (2005). Exposure to ambient air pollution and prenatal and early childhood health effects. Eur J Epidemiol.

[b20-ehp-118-705] Lebret E, Briggs D, Van Reeuwijk H, Fischer P, Smallbone K, Harssema H (2000). Small area variations in ambient NO_2_ concentrations in four European areas. Atmos Environ.

[b21-ehp-118-705] Lestou VS, Kalousek DK (1998). Confined placental mosaicism and intrauterine fetal growth. Arch Dis Child Fetal Neonatal Ed.

[b22-ehp-118-705] Liu S, Krewski D, Shi Y, Chen Y, Burnett RT (2007). Association between maternal exposure to ambient air pollutants during pregnancy and fetal growth restriction. J Expo Sci Environ Epidemiol.

[b23-ehp-118-705] Maisonet M, Correa A, Misra D, Jaakkola JJ (2004). A review of the literature on the effects of ambient air pollution on fetal growth. Environ Res.

[b24-ehp-118-705] Mannes T, Jalaludin B, Morgan G, Lincoln D, Sheppeard V, Corbett S (2005). Impact of ambient air pollution on birth weight in Sydney, Australia. Occup Environ Med.

[b25-ehp-118-705] Nathanielsz PW (2000). Fetal programming: how the quality of fetal life alters biology for a lifetime. Neoreviews.

[b26-ehp-118-705] Nethery E, Brauer M, Janssen P (2009). Time-activity patterns of pregnant women and changes during the course of pregnancy. J Expo Sci Environ Epidemiol.

[b27-ehp-118-705] Owen P, Burton K, Ogston S, Khan KS, Howie PW (2000). Using unconditional and conditional standard deviation scores of fetal abdominal area measurements in the prediction of intrauterine growth restriction. Ultrasound Obstet Gynecol.

[b28-ehp-118-705] Parker JD, Woodruff TJ, Basu R, Schoendorf KC (2005). Air pollution and birth weight among term infants in California. Pediatrics.

[b29-ehp-118-705] Pedersen NG, Figueras F, Wojdemann KR, Tabor A, Gardosi J (2008). Early fetal size and growth as predictors of adverse outcome. Obstet Gynecol.

[b30-ehp-118-705] Perni SC, Chervenak FA, Kalish RB, Magherini-Rothe S, Predanic M, Streltzoff J (2004). Intraobserver and interobserver reproducibility of fetal biometry. Ultrasound Obstet Gynecol.

[b31-ehp-118-705] Pinheiro JC, Bates DM (2000). Mixed-Effects Models in S and S-PLUS.

[b32-ehp-118-705] Ribas-Fitó N, Ramón R, Ballester F, Grimalt J, Marco A, Olea N (2006). Child health and the environment: the INMA Spanish study. Paediatr Perinat Epidemiol.

[b33-ehp-118-705] Rich D, Demissie K, Lu SE, Kamat L, Wartenberg D, Rhoads GG (2009). Ambient air pollutant concentrations during pregnancy and the risk of fetal growth restriction. J Epidemiol Community Health.

[b34-ehp-118-705] Ritz B, Wilhelm M (2008). Ambient air pollution and adverse birth outcomes: methodologic issues in an emerging field. Basic Clin Pharmacol Toxicol.

[b35-ehp-118-705] Royston P (1995). Calculation of unconditional and conditional reference intervals for foetal size and growth from longitudinal measurements. Stat Med.

[b36-ehp-118-705] Schwartz J (2004). Air pollution and children’s health. Pediatrics.

[b37-ehp-118-705] Sinclair KD, Lea RG, Rees WD, Young LE (2007). The developmental origins of health and disease: current theories and epigenetic mechanisms. Soc Reprod Fertil.

[b38-ehp-118-705] Slama R, Darrow L, Parker J, Woodruff TJ, Strickland M, Nieuwenhuijsen M (2008a). Meeting report: atmospheric pollution and human reproduction. Environ Health Perspect.

[b39-ehp-118-705] Slama R, Khoshnood B, Kaminski M (2008b). How to control for gestational age in studies involving environmental effects on fetal growth [Letter]. Environ Health Perspect.

[b40-ehp-118-705] Slama R, Morgenstern V, Cyrys J, Zutavern A, Herbarth O, Wichmann HE (2007). Traffic-related atmospheric pollutants levels during pregnancy and offspring’s term birth weight: a study relying on a land-use regression exposure model. Environ Health Perspect.

[b41-ehp-118-705] Slama R, Thiebaugeorges O, Goua V, Aussel L, Sacco P, Bohet A (2009). Maternal personal exposure to airborne benzene and intra-uterine growth. Environ Health Perspect.

[b42-ehp-118-705] Šrám RJ, Binková B, Dejmek J, Bobak M (2005). Ambient air pollution and pregnancy outcomes: a review of the literature. Environ Health Perspect.

[b43-ehp-118-705] Westerway SC, Davison A, Cowell S (2000). Ultrasonic fetal measurements: new Australian standards for the new millennium. Aust N Z J Obstet Gynaecol.

[b44-ehp-118-705] Wilhelm M, Ritz B (2005). Local variations in CO and particulate air pollution and adverse birth outcomes in Los Angeles County, California, USA. Environ Health Perspect.

[b45-ehp-118-705] Woodruff TJ, Parker JD, Darrow LA, Slama R, Bell ML, Choi H (2009). Methodological issues in studies of air pollution and reproductive health. Environ Res.

